# Transformers for Neuroimage Segmentation: Scoping Review

**DOI:** 10.2196/57723

**Published:** 2025-01-29

**Authors:** Maya Iratni, Amira Abdullah, Mariam Aldhaheri, Omar Elharrouss, Alaa Abd-alrazaq, Zahiriddin Rustamov, Nazar Zaki, Rafat Damseh

**Affiliations:** 1 Department of Computer Science and Software Engineering United Arab Emirates University Al Ain United Arab Emirates; 2 Weill Cornell Medicine-Qatar Doha Qatar

**Keywords:** 3D segmentation, brain tumor segmentation, deep learning, neuroimaging, transformer

## Abstract

**Background:**

Neuroimaging segmentation is increasingly important for diagnosing and planning treatments for neurological diseases. Manual segmentation is time-consuming, apart from being prone to human error and variability. Transformers are a promising deep learning approach for automated medical image segmentation.

**Objective:**

This scoping review will synthesize current literature and assess the use of various transformer models for neuroimaging segmentation.

**Methods:**

A systematic search in major databases, including Scopus, IEEE Xplore, PubMed, and ACM Digital Library, was carried out for studies applying transformers to neuroimaging segmentation problems from 2019 through 2023. The inclusion criteria allow only for peer-reviewed journal papers and conference papers focused on transformer-based segmentation of human brain imaging data. Excluded are the studies dealing with nonneuroimaging data or raw brain signals and electroencephalogram data. Data extraction was performed to identify key study details, including image modalities, datasets, neurological conditions, transformer models, and evaluation metrics. Results were synthesized using a narrative approach.

**Results:**

Of the 1246 publications identified, 67 (5.38%) met the inclusion criteria. Half of all included studies were published in 2022, and more than two-thirds used transformers for segmenting brain tumors. The most common imaging modality was magnetic resonance imaging (n=59, 88.06%), while the most frequently used dataset was brain tumor segmentation dataset (n=39, 58.21%). 3D transformer models (n=42, 62.69%) were more prevalent than their 2D counterparts. The most developed were those of hybrid convolutional neural network-transformer architectures (n=57, 85.07%), where the vision transformer is the most frequently used type of transformer (n=37, 55.22%). The most frequent evaluation metric was the Dice score (n=63, 94.03%). Studies generally reported increased segmentation accuracy and the ability to model both local and global features in brain images.

**Conclusions:**

This review represents the recent increase in the adoption of transformers for neuroimaging segmentation, particularly for brain tumor detection. Currently, hybrid convolutional neural network-transformer architectures achieve state-of-the-art performances on benchmark datasets over standalone models. Nevertheless, their applicability remains highly limited by high computational costs and potential overfitting on small datasets. The heavy reliance of the field on the brain tumor segmentation dataset hints at the use of a more diverse set of datasets to validate the performances of models on a variety of neurological diseases. Further research is needed to define the optimal transformer architectures and training methods for clinical applications. Continuing development may make transformers the state-of-the-art for fast, accurate, and reliable brain magnetic resonance imaging segmentation, which could lead to improved clinical tools for diagnosing and evaluating neurological disorders.

## Introduction

**Figure 1:**
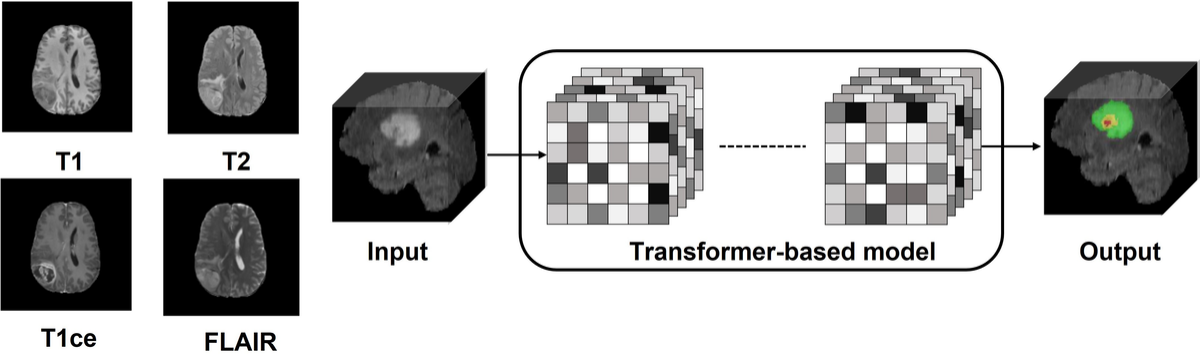
Magnetic resonance imaging modalities of brain tumor. FLAIR: fluid-attenuated inversion recovery; T1: T1-weighted imaging; T2: T2-weighted imaging; T1ce: T1-weighted contrast-enhanced imaging.

Neuroimaging refers to the visualization of the structure and function of the brain. It is one of the most important tools in the understanding of different neurological disorders. Generally, neuroimages can be obtained using 3 principal imaging modalities, where each modality shows the complexities of the brain from a different perspective. Of the 3, magnetic resonance imaging (MRI) is still the most frequently used due to high contrasting ability of brain tissues, high spatial resolution, and no risk of radiation exposure [1-3]. For different brain regions to be viewed, multiple MRI sequences are needed, such as T1, T1ce, T2, and fluid-attenuated inversion recovery, as presented in Figure 1 [4]. The second neuroimaging modality is computed tomography (CT), which can produce high-resolution images. On the other hand, it has limited soft tissue characterization, and its radiation risk makes it unsuitable for repetitive use [3,5]. The third neuroimaging modality is positron emission tomography (PET), which integrates nuclear medicine to visualize metabolic activity [2]. PET has high sensitivity, making it effective in detecting metastases, finding abnormalities, and imaging deep structures. However, it has limited resolution, and repeated use causes radiation risk [3,5]. Finding changes in brain tissue through neuroimaging analysis is critical for detecting and monitoring neurological disorders [6] and brain tumors [7]. Segmentation is a useful process in outlining regions of interest in medical images [8], which enables the quantitative assessment of atrophy, growths, and anatomical differences that depict conditions like Alzheimer disease, schizophrenia, and brain tumors among other neurodegenerative diseases [2]. Because of this, segmentation is applied broadly in different medical applications in diagnosis, tissue classification, radiotherapy treatment, and surgical planning [2,9].

Segmentation techniques can be classified into 3 categories: manual, semiautomated, and fully automated. Manual segmentation is the standard for segmentation because it is believed to be the most accurate [10]. The technique, however, is laborious, time-consuming, and subjective, since it depends on human judgment, and this may result in variation in the results because of the different interpretations. Due to this, there has been a great deal of research into automated segmentation techniques to replicate the results from manual segmentation but with a higher level of efficiency and consistency [2]. To do this, 2 early paradigms were used: intensity-based approaches, which include thresholding, edge-detection, and region-based [8], and traditional machine learning paradigms, including support vector machine, k-nearest neighbor clustering, and random forest [8,11]. Each of these methods has been applied in 1 or more ways, but their applicability and performance within the task of image segmentation remain limited [11,12]. Since then, deep learning (DL) methods have transformed medical imaging applications and became a strong alternative to classical techniques.

DL is a subclass of machine learning that involves artificial neural networks with multiple layers. These networks are designed to progressively learn hierarchical representations and features of data, which both eliminates the need for manual feature engineering [2] and enables the extraction of complicated patterns from large datasets [13]. Different DL architectures have been used for medical image segmentation, but the most widely used and popular one is convolutional neural networks (CNNs), which have achieved state-of-the-art performances in different medical imaging tasks, including segmentation [12,14]. U-Net [15] is another notable model that was specially designed for biomedical image segmentation and has produced very good results in its field [16-18]. Some other notable models include SegNet [19], ResNet [20], DenseNet [21], 3D-ConvNet [22], and DeepLab [23]. These models have served as a solid foundation for the imaging field and have resulted in a plethora of variants, each developed for specific imaging modalities, anatomical structures, and segmentation tasks. Transformers [24] are a type of neural network architectures that mainly rely on self-attention mechanisms. They were first proposed in 2017 and have since yielded state-of-the-art results in the field of natural language processing [25]. More recently, transformers have also shown success when applied to a wide array of computer vision tasks, one of which is segmentation [26]. Although CNNs have achieved impressive performances in image-related tasks, they may not capture global and long-range dependencies well due to the small kernel size [26,27].

Transformers have recently gained popularity in imaging due to their self-attention mechanism, which can model these long-range dependencies—especially useful in brain segmentation [27]. The great success of transformers has motivated the construction of vision transformers (ViT) [28], which forego the use of convolutional layers and rely instead on a multihead self-attention mechanism [13,29]. This architecture divides an image into fixed-size patches, linearly embeds them, and processes them through a transformer network, thereby allowing it to model long-range dependencies with reduced inductive bias [27,30]. Recently, ViT architectures specifically designed for medical image segmentation have been explored and resulted in models like TransUnet [31] and Swin-UNet [32] for general-purpose use and models like TransBTS [33] and Swin-UNETR [34] with the backbone for brain tumor segmentation [29,30]. Special study deserves transformer use in neuroimaging, as the structures of the brain are complicated. Neural networks based on transformers can model long-range dependencies and spatial relationships of the brain images [27], which is very important in brain segmentation.

Although transformers have shown very promising results in many medical imaging tasks, their use in neuroimaging segmentation remains an evolving field that had not been systematically reviewed. Existing literature reviews have either examined the use of transformers for general medical image segmentation without focusing specifically on brain segmentation [25,29,30] or have reviewed brain segmentation techniques using various DL methods without emphasizing the role of transformers [2,13]. One more difference that exists between this review and others is the focus on applying transformers to neuroimage segmentation, which is a central task in neurological disorder diagnosis and treatment. For example, compared with more general surveys such as those by Shamshad et al [29] and Xiao et al [26], which address a wide range of tasks or organ systems, our work specifically focuses on the unique challenges and developments within brain image segmentation. Thus, this scoping review will seek to fill the gap by focusing solely on transformer applications in neuroimage segmentation, an area of paramount importance for the diagnosis and treatment of neurological disorders. A scoping review on this topic is appropriate because the application of transformers in this area is relatively new and fast-developing; hence, it allows for comprehensively mapping the current research landscape and identifying knowledge gaps.

The main purpose of this scoping review is to synthesize and critically evaluate the existing literature on the use of different transformer models for neuroimaging segmentation. This review aims at summarizing the types of transformer models applied, their performance, applications in various neurological conditions and imaging modalities, limitations of the current literature, and highlighting the existing gaps in research.

## Methods

### Study Design

The approach of this scoping review follows the PRISMA-ScR (Preferred Reporting Items for Systematic Reviews and Meta-Analyses extension for Scoping Reviews) guidelines (Multimedia Appendix 1) [35]. Our primary research question was “What are the current applications, performance, and limitations of transformer models in neuroimaging segmentation?”

The goal was the extraction of key themes within recent literature related to transformer use in neuroimaging segmentation that will guide future research and clinical applications. Only the literature starting from 2019 was considered, since the rapid evolution in the development of transformer models for medical imaging is a key recent development in this field.

We defined transformer models as DL architectures relying on self-attention mechanisms, capable of processing sequential data and capturing long-range dependencies. From the neuroimaging perspective, we considered those studies where these models were applied to different modalities of brain imaging, focusing on MRI due to its prevalence in neurological diagnostics.

Our review process followed a systematic search in 4 major databases: Scopus, IEEE Xplore, PubMed, and ACM Digital Library. We present a comprehensive review of methodologies, results, strengths, and limitations of the included studies to derive useful insights that bridge technical developments with their implications in the clinical domain of neuroimage segmentation.

### Search Strategy

Studies were retrieved on May 22, 2023, through searching the following databases: IEEE Xplore, ACM Digital Library, Scopus, and PubMed. The search was limited to 5 years, from 2019 to 2023, to prioritize recent research and consisted of search queries related to transformers such as “transformer,” “deep learning,” and “self-attention”; queries related to neuroimaging such as “brain,” “neuroimaging,” “MRI,” “CT,” and “PET”; and queries related to the medical field such as “health care,” “medical,” “health.”

### Study Eligibility Criteria

This review only included papers whose primary purpose was on the use of transformers for the segmentation of neuroimages. Our search included journal papers, conference papers, and dissertations that focused on applying transformer models to imaging scans (eg, MRI and CT) of the human brain. We excluded all studies that (1) used transformers for the segmentation of nonneuroimaging, raw brain signals, or electroencephalogram data; (2) were not in English, review papers, conference abstracts, preprints, protocols, and conference abstracts; (3) focused on neuroimaging tasks other than segmentation (eg, classification and prediction); and (4) were published before 2019.

### Study Selection

For this review, we used a 3-step study selection process. First, we used EndNote (Clarivate) to remove duplicate studies returned by our initial search. Next, 3 independent reviewers (MI, AA, and MA) screened the titles and abstracts of the remaining papers to exclude irrelevant studies. We then obtained full texts of the studies that passed the initial screening, and the same 3 reviewers (MI, AA, and MA) examined them against our predefined inclusion criteria. Any disagreements between reviewers during the screening processes were resolved through in-person discussions until a consensus was reached.

### Data Collection

The data extraction for this review is done in Microsoft Excel by 2 independent groups of 2 reviewers (MI and AA and MA and OE) to share the workload for extraction and resolve conflicts between the groups. Disagreements in data extraction were resolved through consensus during face-to-face discussions. Data extractions fall into 3 broad categories: study characteristics, neuroimaging acquisition, and transformer features.

Synthesis of included data was done using a narrative approach. Descriptive texts, tables, and figures describe and show the summary and characteristics of the data. Microsoft Excel was used to manage and synthesize the data. First, we depict the characteristics of each included study concerning publication year, type of publication, and country of origin. Then, we describe the neuroimaging acquisition of these studies by including the imaging modality, dataset, dataset accessibility, and neurological condition. Finally, it explains the transformer architecture of the included studies: the number of parameters, transformer type, hybrid component, and the training and evaluation methodology used along with loss function, optimizer, and metrics.

### Ethical Considerations

This scoping review synthesized and analyzed publicly available research studies. No direct human participant research was conducted; therefore, approval from an institutional review board or a research ethics committee was unnecessary. There was no collection, use, or dissemination of personal data from human participants in this study. Data extracted and analyzed in this review had been sourced from published studies, previously subjected to ethical review processes as part of their original publication. The review followed ethics in research practices, in that the representation of the included studies was true to form, and the methodology for the selection of studies and extraction was transparent. No individual participant data were accessed or reported in this review; hence, privacy and confidentiality were ensured. Since this study did not involve direct contact with human participants, issues regarding informed consent and protection of privacy and compensation of participants were therefore not relevant in this study. No images or supplementary material showing identifiable information of any individual were used in this review.

## Results

### Overview

**Figure 2:**
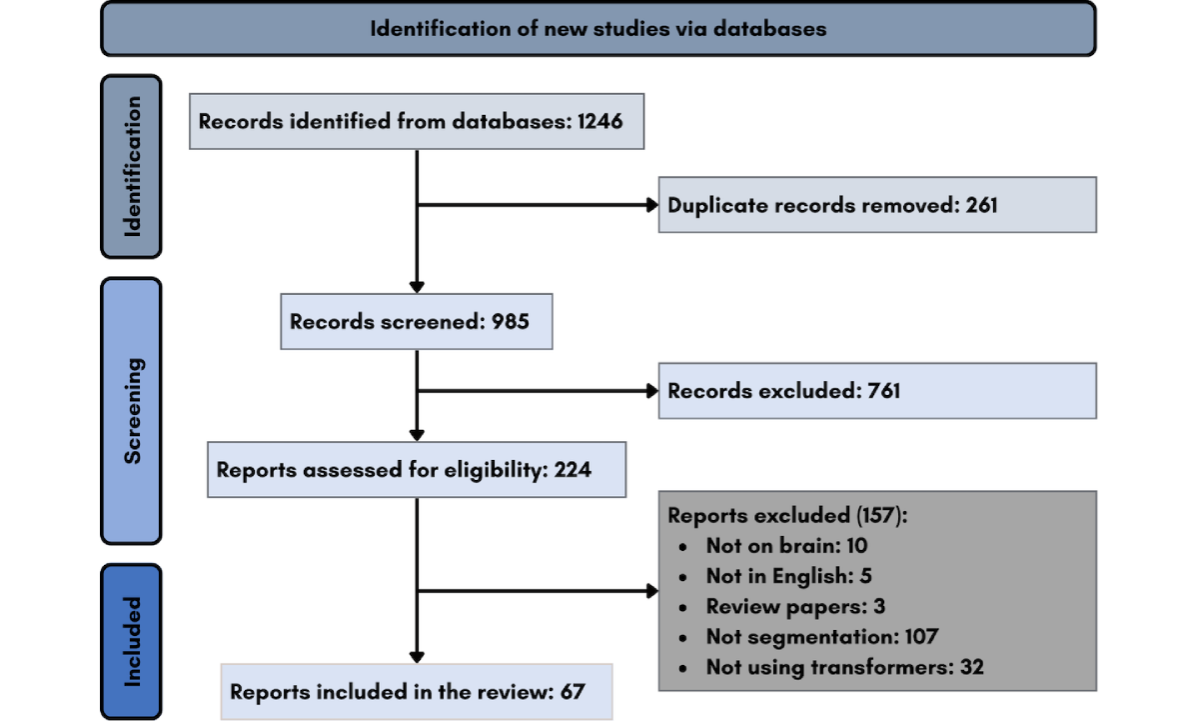
PRISMA-ScR (Preferred Reporting Items for Systematic Reviews and Meta-Analyses Extension for Scoping Reviews) flowchart of the study selection process.

A total of 1246 publications were retrieved from the initial search of the selected databases. In the first round of screening, 261 duplicates were identified and removed through the use of EndNote X9, leaving 985 publications remaining. In the second round, 761 publications were excluded through the analysis of their titles and abstracts against our predefined inclusion or exclusion criteria. The remaining 224 publications continued to the third round of screening, which included a detailed full-text read-through and resulted in the exclusion of 156 publications. Of the 1246 initial publications, only 68 studies met our criteria and were thereby included in this review. Figure 2 depicts the full screening process in more detail.

### Characteristics of the Included Studies

Table 1 depicts the characteristics and metadata of each included study, including the publication year, country, and type. Included studies ranged between 2019 and 2023. Over half of the included studies were published in 2022, followed by 32.84% (n=22) in 2023. Studies included peer-reviewed papers (n=48, 71.64%) and conference papers (n=19, 29.36%). The included studies spanned a total of 13 countries, with China being by far the largest contributor in this domain, representing 68.66% (n=46) of the total studies. Following China, we can find the United States (n=5, 7.46%), the United Kingdom (n=4, 5.97%), and India (n=3, 4.48%), with other countries contributing 1 paper apiece.

**Table 1:** Characteristics of the studies used in this review, including the year, type, and country of publication.

Features		Studies, n (%)	References
**Year of publication**			
	2023	22 (32.84)	[36-57]
	2022	34 (50.75)	[58-91]
	2021	10 (14.93)	[33,34,92-99]
	2019	1 (1.49)	[100]
**Type of publication**			
	Journal paper	48 (71.64)	[34,36-51,53-76,80,83,86,87,91,92,96]
	Conference paper	19 (29.36)	[33,52,77-79,81,82,84,85,88-90,93-95,97-100]
**Country of publication**			
	China	46 (68.66)	[33,37-42,44,46-48,50,54-67,69,72,74-77,79-84,86,88,90-94,96,99]
	United States	5 (7.46)	[34,52,85,92,100]
	United Kingdom	4 (5.97)	[53,68,71,97]
	India	3 (4.48)	[49,95,98]
	Other	9 (13.43)	[36,43,45,51,70,73,78,87,89]

### Neuroimaging Acquisition and Neurological Condition

Table 2 depicts the different imaging modalities used, the datasets used, and the different neurological conditions across the included studies. The included studies included a range of 6 different modalities, with MRI being by far the most common with 88% (n=59), followed by CT with 10.45% (n=7), and the remaining modalities with 1 each. Over half of the included studies used only 1 dataset (n=40, 59.70%) for training and evaluation purposes, followed by 23.88% (n=16) using 2 datasets. Of the 44 unique datasets used across the included studies, 70.45% (n=31) are public or open-source datasets, and 29.55% (n=13) are private datasets obtained directly from medical institutions. Regarding the public dataset category, the brain tumor segmentation dataset (BraTS dataset) is by far the most widely used, with 58.21% (n=39) of total studies using its variants (including BraTS 2015, 2017, 2018, 2019, 2020, and 2021), followed by Medical Segmentation Decathlon and low-grade glioma-Kaggle with 5.97% (n=4) each. The main neurological condition of the included studies was the segmentation of brain tumors, with 71.64% (n=48) of studies conducting research specifically in this area.

**Table 2:** Description of features used in the included studies, including modalities, datasets, dataset types, and neurological conditions.

Features		Studies, n (%)	References
**Imaging modality**			
	MRI^a^	59 (88.06)	[33,34,36-51,54-69,71-85,87,88,90,92,93,95-99]
	CT^b^	7 (10.45)	[52,53,71,75,86,89,94]
	PET^c^	1 (1.49)	[73]
	Interventional ultrasound	1 (1.49)	[70]
	Electron microscopy	1 (1.49)	[100]
	Digital subtraction angiography	1 (1.49)	[91]
**Number of datasets used**			
	1	40 (59.7)	[34,36,39,40,42-50,53,57,63-65,69,70,74,77,78,80,83-88,90-95,97-100]
	2	16 (23.88)	[33,37,38,41,51,56,58,59,61,67,68,73,76,82,89,96]
	3+	10 (14.93)	[54,55,60,62,66,71,72,75,79,81]
	Not mentioned	1 (1.49)	[52]
**Dataset accessibility**			
	Public	31 (70.45)	—^d^
	Private	13 (29.55)	—
**Dataset**			
	Public: BraTS^e^	39 (58.21)	[33,34,37,40-42,44-46,48,50,51,54,56,60-62,64-66,68,69,71,72,74,76,77,79-83,90,92,93,95,97-99]
	MSD^f^	4 (5.97)	[39,62,85,88]
	iseg-2017	3 (4.48	[58,59,96]
	ADNI^g^	2 (2.99)	[43,73]
	MRBrainS^h^	2 (2.99)	[59,96]
	LGG^i^-Kaggle	4 (5.97)	[49,63,66,84]
	ISLES^j^	3 (4.48)	[51,54,75]
	WMH^k^	3 (4.48)	[71,78,81]
	Other	12 (17.91)	[55,58,68,70,71,73,75,81,87,89,91,100]
	Private	9	[36,38,47,53,57,67,82,86,94]
	Not stated	1 (1.49)	[52]
**Neurological condition**			
	Brain tumor	48 (71.64)	[33,34,37-42,44-46,48-51,54,56,60-72,74,76,77,79-85,88,90,92,93,95,97-99]
	Ischemic stroke	4 (5.97)	[51,52,75,94]
	Alzheimer disease	3 (4.48)	[43,73,81]
	Parkinson disease	2 (2.99)	[36,57]
	Intracerebral hemorrhage	3 (4.48)	[53,86,89]
	Intracranial aneurysms	1 (1.49)	[91]
	Autism	1 (1.49)	[55]
	Brain lesions	1 (1.49)	[78]
	Healthy brain	6 (8.96)	[47,58,59,87,96,100]

^a^MRI: magnetic resonance imaging.

^b^CT: computed tomography.

^c^PET: positron emission tomography.

^d^Not available.

^e^BraTS: brain tumor segmentation dataset.

^f^MSD: Medical Segmentation Decathlon.

^g^ADNI: Alzheimer’s Disease Neuroimaging Initiative.

^h^MRBrainS: magnetic resonance brain image segmentation.

^i^LGG: low-grade glioma.

^j^ISLES: ischemic stroke lesion segmentation.

^k^WMH: white matter hyperintensities.

### Transformer-Based Techniques Types, Training Parameters, and Evaluation

**Figure 3:**
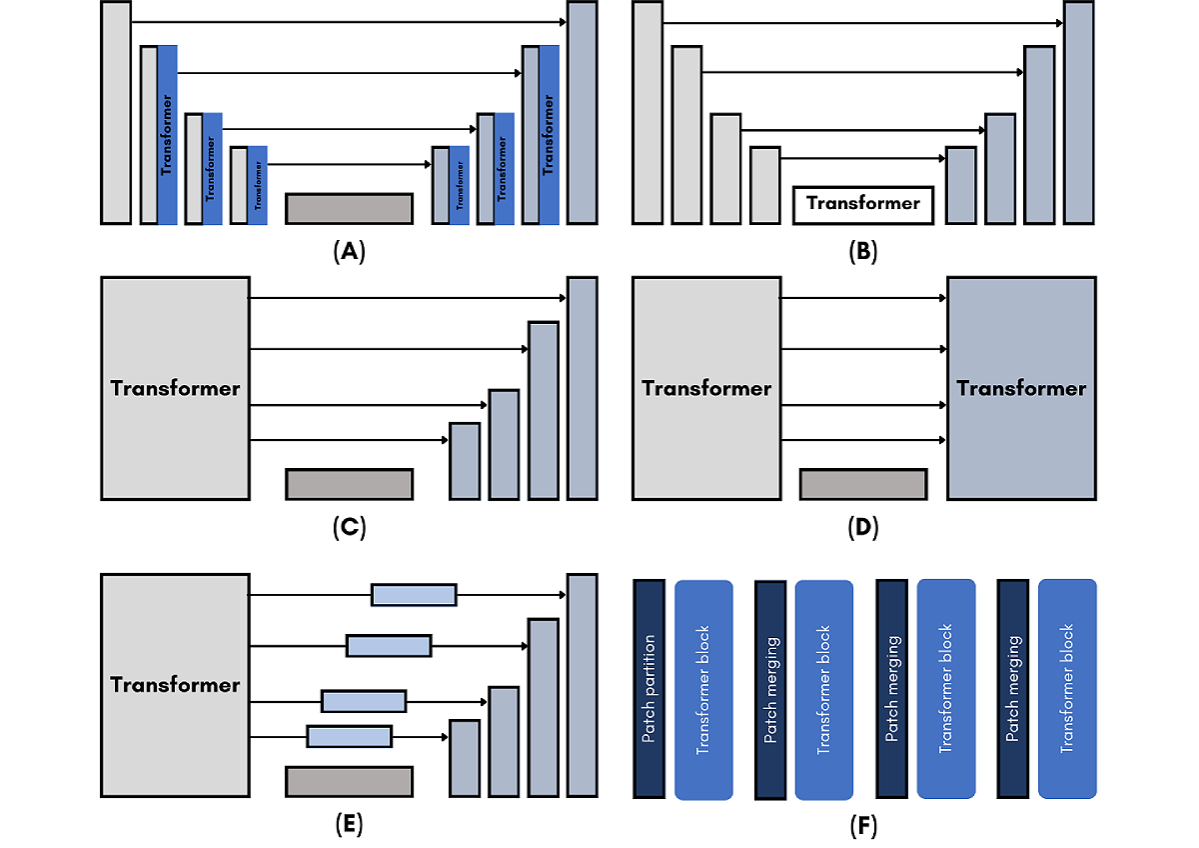
Various transformer-based architectures used for neuroimage segmentation. (A) U-Net+transformer, (B) TransBTS, (C) TransUNet, (D) SwinUnet, (E) UNETR, and (F) transformer.

The proposed neuroimage segmentation techniques used various artificial intelligence (AI) techniques. In this review, we focused on the deep transformer–based techniques that have gained more attention recently. From the proposed models, we can find transformer-based, CNN with transformer-based, and generative adversarial network with transformer-based techniques. At the same time, the methods based on TransBTS, TransUNet, SeinUNet, and U-Net with transformer are the most used models for neuroimage segmentation. Figure 3 illustrates these models in terms of architecture. Table 3 depicts the characteristics of transformer models used within the included studies. From Table 3, we can find that 58.21% (n=39) of the included studies did not explicitly report the number of parameters of their proposed models. Of the studies that did, however, the majority of the transformer models proposed had between 20 and 40 million parameters (n=10, 14.93%), followed by 1 and 19 million (n=8, 11.94%). A majority of studies implemented a 3D segmentation network (n=42, 62.69%), with 37.31% (n=25) being 2D. An overwhelming 85.07% (n=57) of included studies proposed transformer models that are hybrid, with only 14.93% (n=10) of them being standalone transformer models. ViT was the most used transformer architecture, with 55.22% (n=37) of studies using it as its main component. Another significant transformer model is the Swin transformer, with 20.89% (n=14), followed by TransUnet, with 5.97% (n=4). Of the 57 hybrid transformer models, 55 (96.49%) studies opted for a combination of CNN with their transformer, and of those 55 CNN-transformer models, 56.36% (n=31) were U-Net based, and 9.09% (n=5) were ResNet based. Both generative adversarial network (n=2, 3.51%) and autoencoders (n=2, 3.51%) were also combined with transformers.

**Table 3:** Proposed methods based on the weight of the models, type of data used, and type of transformer technique used.

Features		Studies, n (%)	References
**Number** **of** **parameters** **(in millions)**			
	1-19	8 (11.94)	[41,42,45,51,68,76,79,82]
	20-39	10 (14.93)	[33,37,39,56,58,62,63,65,69,88]
	40-59	4 (5.97)	[42,52,60,86]
	60-100	3 (4.48)	[34,47,85]
	100-120	3 (4.48)	[53,80,97]
	120+	2 (2.99)	[42,54]
	Not mentioned	39 (58.21)	[36,38,40,43,44,46,48-50,55,57,59,61,64,66,67,70-75,77,78,81,83,84,87,89-96,98-100]
**Dimensionality**			
	2D	25 (37.31)	[40,43,45,48,49,53,54,58,63,70,75,77,78,84,86,88,89,91,94,99,100]
	3D	42 (62.69)	[33,34,36-39,41,42,44,46,47,50-52,55-57,59-62,64,69,71-74,76,79-83,85,87,90,92,93,95-98]
**Transformer model**			
	Standalone	10 (14.93)	[38,42,54,55,61,75,78,82,89,100]
	Hybrid	57 (85.07)	[33,34,36,37,39-41,43-53,56-60,62-74,76,77,79-81,83-88,90-99]
**Type** **of** **transformer**			
	ViT^a^	37 (55.22)	[33,36,38,40,44,46,47,49-55,57-60,65,66,71,73,75,81-83,85,87,88,90,91,93,95-99]
	Swin	14 (20.89)	[34,37,42,43,56,62,67-69,72,74,76,77,89]
	SwinUnet	2 (2.99)	[61,78]
	TransUnet	4 (5.97)	[45,70,84,94]
	TransBTS	2 (2.99)	[64,92]
	Other	8 (11.94)	[39,41,48,63,79,80,86,100]
**Type** **of** **hybrid** **component**			
	CNN^b^	55 (96.49)	[33,34,36,37,39-41,43-53,56-60,62-70,72-74,76,77,79,83-88,90-99]
	U-Net	31 (56.36)	[34,36,37,40,43,45,46,48-51,53,56,57,60,62-64,70,76,83-86,88,90-94,97]
	ResNet	5 (9.09)	[59,60,66,67,94]
	GAN^c^	2 (3.51)	[43,60]
	Autoencoder	2 (3.51)	[71,81]

^a^ViT: vision transformer.

^b^CNN: convolutional neural network.

^c^GAN: generative adversarial network.

Table 4 depicts the loss function used, the optimizer used, and the different evaluation methods used across each included study. The loss function was not mentioned in 11.94% (n=8) of the included studies. Of the studies that mentioned it, the most popular loss function is a combination of cross-entropy and Dice loss with 40.30% (n=27) of included studies, followed by Dice loss with 19.40% (n=13). Adam is the most used optimizer, with 47.76% (n=32) of included studies using it, followed by AdamW at 14.92% (n=10). However, the optimizer was not mentioned in 22.39% (n=15) of studies. In terms of evaluation, over half of the included studies used at least two evaluation metrics (n=34, 50.75%), followed by 1 metric (n=11, 16.42%). Of these evaluation metrics, the Dice score is by far the most used, with 94.03% (n=63) of all studies using it, followed by HD95, 52.24% (n=35), and sensitivity, 28.36% (n=19).

**Table 4:** Experimental setups and evaluation for the proposed transformer-based techniques.

Features		Studies, n (%)	References
**Loss** **function1**			
	Dice loss	13 (19.4)	[34,37,38,41,45-49,59,79,80,87]
	Cross-entropy	9 (13.43)	[43,44,50,61,63,65,77,90,96]
	Dice cross-entropy	27 (40.3)	[39,40,42,51,54,56,57,60,62,69,72,76,78,81-86,91,93-95,97-99]
	Other	10 (14.93)	[33,52,53,55,58,66,68,71,74,89]
	Not mentioned	8 (11.94)	[36,67,70,73,75,88,92,100]
**Optimizer**			
	Adam	32 (47.76)	[33,37,40,41,44,49,53,55,60-62,65-69,72,75,76,78,80,86-88,91-93,95,97-99]
	AdamW	10 (14.92)	[42,48,51,52,56,74,81,82,85,89]
	SGD^a^	7 (10.45)	[47,50,54,57,83,94,97]
	Ranger	2 (2.99)	[38,79]
	RMSprop^b^	1 (1.49)	[63]
	Apollo	1 (1.49)	[64]
	Not mentioned	15 (22.39)	[34,36,39,43,45,46,58,59,70,71,73,84,90,96,100]
**Evaluation** **metrics**			
	Dice score	63 (94.03)	[33,34,36-42,44-66,68-72,74-99]
	HD95^c^	35 (52.24)	[33,34,40-42,44-48,50,51,54,56-58,61,62,64,65,72,74,76,78,79,81,82,85,90,92,93,95,97-99]
	Recall or sensitivity	19 (28.36)	[43,44,46,47,53-55,57,60,64,69,75,83,86,87,89,90,93,94]
	IoU^d^	12 (17.91)	[45,49,52-54,63,66,68,77,87,89,91]
	Precision	10 (11.94)	[43,44,47,53,55,60,75,87,89,90]
	Accuracy	6 (8.96)	[43,49,55,67,73,86]
	Specificity	5 (7.46)	[47,86,89,90,93]
	AUC^e^	5 (7.46)	[43,67,71,89,100]
	F-measure	3 (4.48)	[43,78,89]
	Jaccard index	4 (5.97)	[44,53,57,90]
	Other	5 (7.46)	[47,63,66,78,91]

^a^SGD: stochastic gradient descent.

^b^RMSprop: root mean square propagation.

^c^HD95: Hausdorff distance at the 95th percentile.

^d^IoU: Intersection Over Union.

^e^AUC: area under the curve.

### Strengths and Limitations of Transformer-Based Techniques

Transformers have revolutionized the area of neuroimage segmentation by offering unparalleled capabilities in modeling complex features in medical imaging. It has the ability to model both local and global information, which substantially improves the accuracy of segmentation and therefore becomes very useful in various neurological applications. As shown in Table 5, the common strengths of transformer-based techniques include a high mean Dice score, effective fusion of multimodal MRI, and robust performance across diverse and complex datasets. However, these models also have substantial limitations in terms of high computational and memory costs, sensitivity to small areas of tumors, and possible overfitting on smaller datasets.

**Table 5:** Strengths and limitations of common transformer-based techniques.

References		Strengths	Limitations
**ViT^a^**			
	[33,36,38, 40,44,46, 47,49-55, 57-60,65, 66,71,73, 75,81-83, 85,87,88, 90,91,93, 95-99]	Effectively models local and global features in 3D MRIb data. High mean Dice score. Demonstrates consistent improvements in segmentation performance. Effective in emphasizing informative brain regions. Uses symmetry of brain structures for improved feature learning. Outperforms state-of-the-art SSLc methods and medical image segmentation models on benchmarks. Incorporates gradient-based scoring for attentive reconstruction. Effective multimodal MRI fusion. Enhanced long-term dependencies within individual modalities. Complementary contextual information among modalities.	Computationally intensive. High computational and memory cost. Memory constraints when lowering patch resolution. Complexity in integrating CNNd and transformer features. Sensitivity to small tumor areas in LGGe. Overlap in feature dimensions between CNN and transformer branches. Misclassification of voxels in LGG. Imbalance in dataset affecting performance. Need for extensive validation on more diverse datasets.
**Swin**			
	[34,37,42, 43,56,62, 67-69,72, 74,76,77, 89]	Capable of learning multiscale contextual information, enhancing performance across various tasks. Combines advantages of ViT and CNNs, balancing both local and global feature learning. Maintains high-resolution features, crucial for precise segmentation tasks. Performs efficient tri-level preprocessing, including noise removal improving input quality for better results. Incorporates advantages of 3D Swin transformer, improving performance in 3D medical image analysis.	Slight decrease in performance for specific areas like tumor core segmentation in some instances. ViTs have many parameters and structures, making them complex and resource-intensive. Potential risk of overfitting on smaller datasets due to high model complexity. Existing neural network algorithms may often extract redundant features, reducing overall efficiency. Limited exploration in preprocessing and postprocessing techniques, which might enhance model performance further.
**SwinUnet**			
	[61,78]	Improves the efficiency of using limited labeled data. Competitive performance in Dice score, Hausdorff distance, and other segmentation metrics.	Requires verification of the improvement with diverse and larger datasets. Lower recall and F1-scores compared to other CNN-based methods.
**TransUnet**			
	[45,70,84, 94]	High effectiveness in model design. Combines the strengths of U-Net and transformer models. Achieves higher Dice scores compared to U-Net and transformer. Effectively learns global context features in images.	Some models within the study showed poor performance. Cross-application limitations in certain scenarios. High data requirements for effective training. Limited dataset size affecting generalization.
**TransBTS**			
	[64,92]	Residual basis blocks reduce feature loss and enhance feature extraction. Combines CNN and transformer for improved segmentation performance, leveraging both local and global information. Attention mechanisms enhance the model’s ability to focus on relevant features, improving accuracy.	Increased network depth leads to higher parameter counts, increasing computational requirements. May struggle with unseen patterns in the testing phase, affecting performance robustness.
**Other transformer types**			
	[39,41,48, 63,79,80, 86,100]	Effectively capture both local and global context in medical images. Enhance segmentation accuracy. Robust across diverse and complex datasets. Improve semantic information representation. Combine multiresolution information effectively.	High computational complexity. Significant memory use. Requires substantial hardware resources. Extensive data preprocessing is needed. Limited scalability to very large datasets. Sensitive to variations in input data quality.

^a^ViT: vision transformer.

^b^MRI: magnetic resonance imaging.

^c^SSL: self-supervised learning.

^d^CNN: convolutional neural network.

^e^LGG: low-grade glioma.

## Discussion

### Principal Findings

The main purpose of this scoping review is to conduct a thorough investigation into the use of different transformer models in the field of neuroimaging, specifically segmentation. From the gathered data, it is clear that the use of transformers in neuroimaging experienced a great boost in research from 2021 to 2022, with over half of the included studies being published in 2022 compared to only 10 studies in 2021. It is also important to note that for the year 2023, only the studies up to May 22 were included; yet, this constitutes a total percentage of the included studies of 32.84% (n=22) and could very well be even higher when the whole year is considered.

From the studies included in this review, it is clear that MRI is by far the most popular image modality for applying transformer models to neuroimaging segmentation. This can be attributed to how common the use of MRI is in the diagnosis of neurological illnesses, especially for brain tumors [27], wherein it is able to provide functional, structural, and metabolic information [27] through the use of its different modalities (T1, T2, T1ce, and fluid-attenuated inversion recovery). MRI is particularly suitable for neuroimaging segmentation purposes because of the high spatial resolution and soft tissue contrast, both being critical for any form of precise segmentation it exhibits, since it is able to show good detailed visualization of structures in the brain and distinction between different tissues of the brain sizes [1,2].

Another reason for the popularity of MRI in the included studies is the availability of brain MRI scans sourced from the widely used BraTS datasets [101]. This yearly and open-source dataset contains a wide variety of different MRI modalities that are manually annotated, making it a very important resource for developing and benchmarking segmentation methods based on different transformer models. This is why it is no surprise that it is by far the most used dataset in the included studies.

When it comes to neurological conditions, a majority of included studies in this review focused on the use of transformers in brain tumor segmentation. This can be attributed to multiple factors, including the availability of MRI scans from the BraTS dataset that are specifically for brain tumor segmentation. Brain tumors are also highly prevalent among all ages and have a high fatality rate [102], making it a prime area for research into new methods of diagnosis and treatment. In addition, brain tumors are fairly complex and irregular in both location and shape [27], which makes manual segmentation a very tedious and time-consuming process that would benefit greatly from increased research into more automated methods for segmentation. The BraTS dataset is also a factor, as it provides a large variety of MRI scans that are specifically for brain tumor segmentation. Transformers are particularly useful for brain tumor segmentation due to their self-attention mechanism, which allows them to account for different variations in tumor characteristics, such as size and shape, during the segmentation process [26].

Most included studies proposed and developed models with 3D segmentation networks, specifically for 3D imaging data. In terms of neuroimaging, 3D scans are more common in part due to the 3D nature of MRI scans. Since MRI is the most common imaging modality used in neuroimaging, it makes sense that it is preferable to develop models for 3D imaging data in order to avoid the loss of information. Even though 3D models are typically more accurate for 3D imaging segmentation, they are computationally expensive [26], which is why some proposed models in the included studies chose to instead extract 2D slices from 3D imaging data. While this technique is suitable, reducing a 3D scan into 2D slices can lead to a degradation of volume and spatial characteristics native to 3D data [103].

CNN-transformer hybrid models were used far more than standalone transformer models in the included studies, specifically in the form of a U-Net and transformer combination. These combinations capitalize on the strengths of both CNN and transformers while minimizing their weaknesses. CNN is particularly useful in extracting local features and spatial information from the provided scans; however, it often struggles to capture long-range dependencies due to its small kernel size [26,27]. On the other hand, transformers are able to model these long-range dependencies due to their self-attention module, making them very useful for neuroimaging segmentation, especially in the case of brain tumors [27]. This is why most included studies opted to use the use of CNN to capture local features and transformers to capture global features to increase the performance of their models in the task of segmentation [102].

### Research and Practical Implications

This scoping review provides an overview of the available research regarding the use of transformers in the context of neuroimaging segmentation. These findings underline important implications for future research and applications in this area.

It is a notable finding of this review that many studies apply transformers, specifically to brain tumor segmentation, which might hint at the potential of transformers in assisting diagnosis and treatment planning in this field. As shown here, transformers are well-suited for this task. However, further research is needed to assess the real-world clinical usefulness of transformers for brain tumor segmentation. While brain tumors are an important challenge, the focus on this single application at this level would seem indicative of the current lack of large and good-quality datasets in many other big neurological diseases and conditions, such as Alzheimer and Parkinson disease, and strokes. Making publicly available manually annotated datasets of different neurological conditions would motivate new research and developments on the application of transformers in this field. On top of this, the heavy reliance of studies on the BraTS dataset shows that there is a need to diversify datasets in order to validate different models correctly. Most of the included studies favored hybrid use by combining CNN and transformers, which illustrates the complementary strengths of these architectures for neuroimaging segmentation. Success in hybrid techniques shows that further exploration of novel integrations between transformers, CNNs, and other modules could become a promising direction to achieve better performances on more complex medical image analysis problems. Improved accuracy in neuroimaging segmentation, through the ability of transformer models to extract local and global features, allows for more accurate identification of neurological conditions such as brain tumors. This will provide earlier diagnosis and treatment. Moreover, automation with these models will save much time of the clinicians in performing manual segmentation so that they can concentrate on the care of patients and other important tasks. Treatment planning may also be improved with transformer models, where the potential for more accurate and consistent segmentation results helps a lot in this respect. Moreover, these models can also potentially be integrated into clinical workflows without much hassle by developing user-friendly interfaces and collaboration between AI researchers and clinicians to ensure these tools are adopted and effectively used in practice.

### Strengths and Limitations

This scoping review has numerous key strengths with regard to the analysis of transformer applications in neuroimaging segmentation. First, it gives a broad overview of the fast-evolving field by capturing recent works from 2019 through 2023. Second, it allows focusing on current research so that the review reflects the state-of-the-art in transformer applications for medical imaging. It is a systematic approach, covering 4 major databases; hence, wide and comprehensive coverage of the literature reviewed. The inclusion of journal papers and conference papers facilitates a wide view of both consolidated and emergent research. Third, this review gives elaborate insights into various aspects of transformer use in neuroimaging: imaging modality, dataset, neurological condition, and metric for performance evaluation. This level of analysis provides rich information relevant to both researchers and practitioners within the field. Finally, the review’s focus on brain tumor segmentation, while a limitation in some respects, also serves as a strength by providing an in-depth look at transformer applications in a critical area of medical imaging with significant clinical implications.

While this scoping review offers a number of strengths, its limitations need to be acknowledged so as to strike a balance. First, the review was on transformers in neuroimaging segmentation alone, excluding other medical imaging tasks or organs. This narrow focus allows for an in-depth analysis of transformer applications in brain imaging but may not be representative of the full spectrum of use that transformers have seen in medical imaging. This limitation could be reduced by expanding the scope of future reviews to multiple organ systems or imaging tasks, giving a wider look at transformer applications in medical imaging.

Second, the review was focused on studies published in the English language, published from 2019 up to 2023. This narrowing was necessary, as most current works are favored in this novelty area of transformer use in medical imaging. In so doing, this review criterion may have left out important non-English language publications or early applications of transformers. This is likely a limitation in the representation of research trends worldwide. In this respect, future studies can be designed to include more languages, also extending the date range to capture more diverse sets of publications and track the evolution of transformer use in medical imaging over an extended period.

Third, the fact that 58.21% (n=39) of the works included in this review were based on the BraTS dataset introduces a certain bias in the domain toward the segmentation of brain tumors. Though it is a very critical area, it might not be useful to represent transformers completely for other neurological conditions. Future research needs to give more emphasis to developing and publicly releasing manually annotated datasets about more neurological conditions to address this limitation. This will further encourage diverse applications of transformers in neuroimaging and provide a wider understanding of the capability of transformers across different pathologies.

The review demonstrated a high dominance by studies from China, with 46 (68.66%) studies of the total (see Multimedia Appendix 2 for detailed analysis). This aligns with broader publication patterns in AI research where Chinese institutions contribute approximately 40% of global publications. While this distribution reflects documented trends in international research output, future reviews might benefit from more diversified search strategies to ensure comprehensive coverage of global research activities in this field.

Finally, no formal quality or risk-of-bias assessment of the included studies was performed. Although this represents a common approach when it comes to scoping reviews, this limits the degree to which strong conclusions can be drawn about the relative effectiveness of various approaches to transformers. Future systematic reviews or meta-analyses may involve quality assessments to support more robust evidence in terms of the efficacy of transformer models in neuroimaging segmentation.

### Future Directions

These findings point to a variety of promising directions for future research on the application of transformers to neuroimaging segmentation. First, future studies should develop novel integrations between transformers, CNNs, and other advanced modules that will further improve performance for complex medical image analysis tasks. This might be achieved by investigating various hybrid models leveraging the strengths of transformers and more traditional DL methods. Second, the extension of transformer applications to more neurological conditions other than brain tumors, which would allow a wider grasp of the potential capability of transformers across different pathologies. More clinical applications are likely to follow from here. Third, the development of new transformer-based methods or their combination with emerging techniques like diffusion models could further improve efficiency and robustness for both 2D and 3D brain segmentation. Fourth, future studies shall be done to bridge the current limitations in dataset diversity. This may be in creating and publishing manually annotated datasets for a wider range of neurological conditions that can enable transformers to apply to neuroimaging in more diversified ways. Finally, the translation of research findings into clinical practice remains a high unmet need. This transition will require extensive validation of transformer models on diverse, real-world datasets and close collaboration between AI researchers and clinicians. Such collaboration could result in the development of more clinically relevant models and user-friendly interfaces, which would expedite the translation of these advanced technologies into routine clinical practice.

### Conclusions

This scoping review has thoroughly investigated the applications of transformers in neuroimaging segmentation and discovered a highly evolving field with great potential. The results of this paper have shown that transformer models, especially combined with CNNs in hybrid architectures, are also very promising for the task of brain MRI segmentation. Some of the big advantages of transformers include the modeling of long-range dependencies in images through self-attention mechanisms while still being able to perform local feature extraction. Such a combination uniquely allows for more accurate and detailed segmentation in highly complex neurological pathologies, like brain tumors.

There is clearly a trend toward 3D transformer models and hybrid CNN-transformer architectures, dominated by ViT as the variant of transformer used most frequently. These approaches also obtain superior performance on benchmark datasets, such as brain tumor segmentation tasks. However, reliance on the BraTS dataset highlights a requirement for more diverse data sources to ensure that performance could be validated across more multiple neurological conditions.

While this is promising, there are still important issues in the field: high computational costs associated with transformer models, overfitting on smaller datasets, and validation in larger clinical settings. Another issue is the geographical concentration of research output that highlights the need for greater diversity in the origins of studies worldwide to improve the generalizability of findings.

The future prospect of transformer models will unlock the potential that neuroimaging segmentation demands. Refining both architectures and training methods and integration into clinical workflows, transformations may provide state-of-the-art for fast, accurate, and reproducible brain-MRI segmentation, hence advancing clinical diagnosis and evaluation techniques for a better outcome in regard to patients with neurological disorders.

Although transformers have shown great improvement in neuroimaging segmentation, much potential is yet to be realized. Future work will need to be focused on present limitations, the extension of applications across a wider range of neurological conditions, and narrowing the gap between research and clinical practice to ensure that transformers are a valuable and impactful technology in medical imaging analysis.

## Data Availability

All data generated or analyzed during this study are included in this published paper and Multimedia Appendix 1.

## Appendix

Multimedia Appendix 1 PRISMA-ScR (Preferred Reporting Items for Systematic Reviews and Meta-Analyses Extension for Scoping Reviews) checklist.

Multimedia Appendix 2 Significant prevalence of studies published in China.

## Abbreviations

AI: artificial intelligence
BraTS dataset: brain tumor segmentation dataset
CNN: convolutional neural network
CT: computed tomography
DL: deep learning
MRI: magnetic resonance imaging
PET: positron emission tomography
PRISMA-ScR: Preferred Reporting Items for Systematic Reviews and Meta-Analyses Extension for Scoping Reviews
ViT: vision transformer
