# Supplementary material for: Transformers for Neuroimage Segmentation: Scoping Review
Source: J Med Internet Res. 2025 Jan 29;27:e57723. doi: 10.2196/57723 (PMC11822320; doi:10.2196/57723)
Supplement: Multimedia Appendix 2 [file jmir_v27i1e57723_app2.docx]

In our review, we mentioned that there was a major dominance of studies from China (constituting about 69%) in the field of transformers-based neuroimaging segmentation. In this appendix, we will discuss the potential reasons for such dominance and outline that studies published in China are a major contributor to not only in the field of neuroimaging but artificial intelligence (AI) as a whole.

| **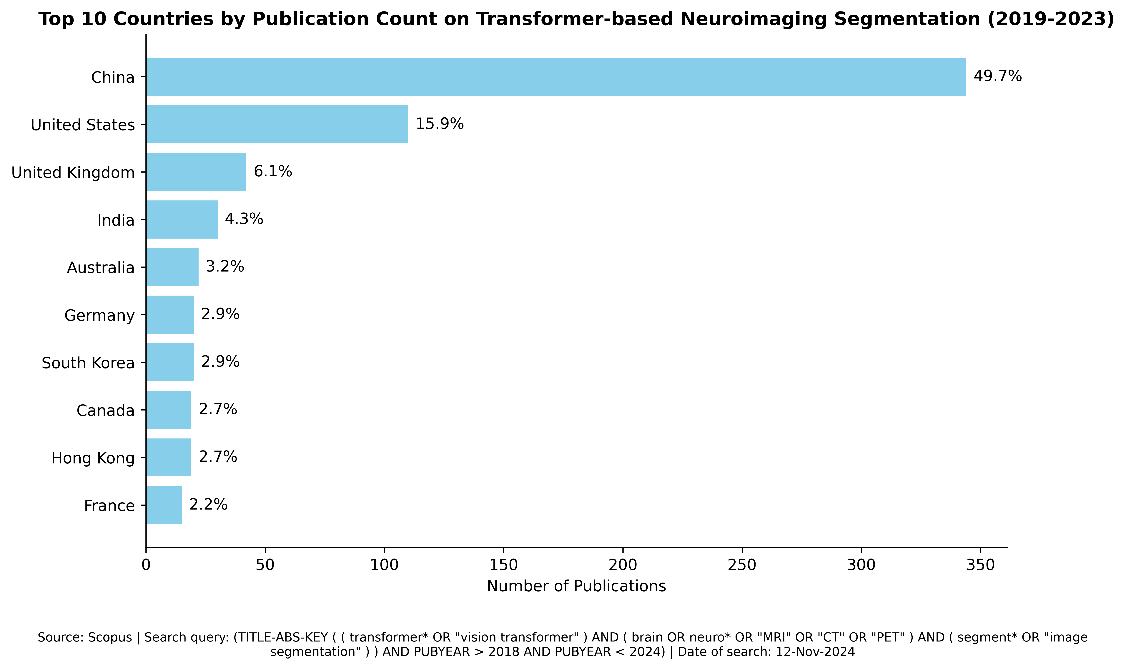**  **(a) Scopus** |
| --- |
| **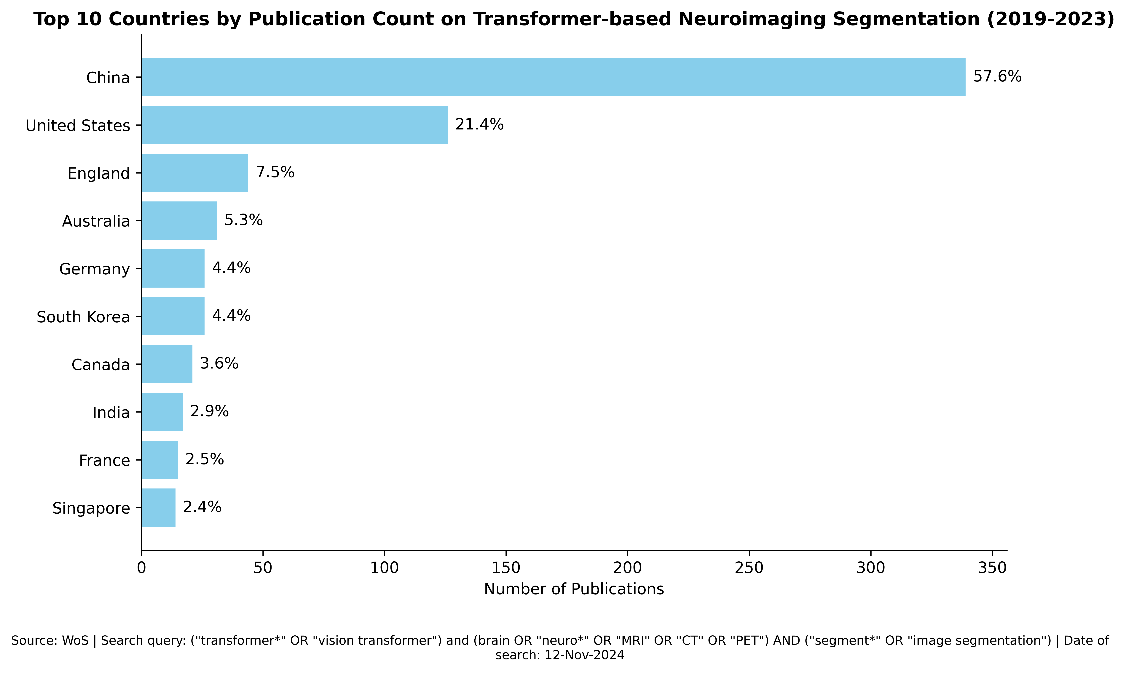**  **(b) Web of Science** |

**Figure S1.** Country-wise Publication Count on Transformer-based Neuroimaging Segmentation between 2019 and 2023 from two major databases. The search terms are provided in the caption of each figure.

Figure S1 illustrates publication count in top ten countries on neuroimaging segmentation using transformer architectures from two major publication databases—Scopus in Figure S1 (a) and Web of Science (WoS) in Figure S1 (b). It is evident from the figures that publications from China are dominating this field as a whole with about 50% in Scopus and 57% in WoS, while the second top publisher—the US—merely accounts for 16% and 21%, respectively. This huge discrepancy of publications from studies published from China indicates that there might be a shift in the research and development (R&D) sector of the country publishing more on the international stage. To further validate this, we will explore China's overall research investment and output patterns across scientific fields, particularly in AI and related technologies.

We can attribute such dominance by studies from China to the country’s substantial investments in R&D and consequently, its abundant research output in all fields, including AI. In 2021, China’s expenditure for R&D reached approximately 2.79 trillion yuan (~ 380 billion USD) accounting for 2.44% of its GDP^[[1]](#footnote-1)^. Notably, this commitment has positioned the publications from China to be major contributors to science and engineering, as by 2022, studies from China accounted for about 27% of global publications, surpassing the US (with 14%)^[[2]](#footnote-2)^. This trend is also reflected in the field of AI, where publications from China accounted for nearly 40% of all AI-related publications in 2021, outpacing other nations by a large margin^[[3]](#footnote-3)^.

From these trends, we can see the reason why studies from China are dominating the field of AI, including neuroimaging segmentation. Other than that, we did not observe any noticeable trends in terms of journals, model (transformer) architectures or other factors, which might have contributed to the high prevalence of papers selected from China in this review paper.

Although our analysis shows a clear picture of China's considerable contribution to the field, publication count alone cannot reflect the whole picture of the research impact. Future studies might want to consider additional bibliometric metrics, such as citation impact, international collaborations, and clinical implementation of the published methods. However, the consistent trend across databases and the broader literature confirms our geographical distribution as representative of the current activity of this domain.

1. **National Bureau of Statistics of China**. (2022, January 27). *Press Release on China’s R&D Expenditure in 2021*. Retrieved from <https://www.stats.gov.cn/english/PressRelease/202201/t20220127_1827065.html>. [↑](#footnote-ref-1)
2. **National Science Foundation & National Science Board**. *Science and Engineering Indicators*. National Center for Science and Engineering Statistics (NCSES), Alexandria, VA. Retrieved from <https://ncses.nsf.gov/pubs/nsb202333/>. [↑](#footnote-ref-2)
3. **Woolston, C**. (2023, August 9). *What China’s leading position in natural sciences means for global research*. *Nature Index*. Retrieved from <https://www.nature.com/articles/d41586-023-02159-7>. [↑](#footnote-ref-3)
